# Different Sources of High Fat Diet Induces Marked Changes in Gut Microbiota of Nursery Pigs

**DOI:** 10.3389/fmicb.2020.00859

**Published:** 2020-05-07

**Authors:** Fei Yang, Shihai Zhang, Min Tian, Jun Chen, Fang Chen, Wutai Guan

**Affiliations:** ^1^Guangdong Provincial Key Laboratory of Animal Nutrition Control, College of Animal Science, South China Agricultural University, Guangzhou, China; ^2^College of Animal Science and National Engineering Research Center for Breeding Swine Industry, South China Agricultural University, Guangzhou, China

**Keywords:** fat type, gut, microbial community, digestibility, nursery pigs

## Abstract

Fat is one of the most important nutrients which provides concentrated energy and essential fatty acids. High fat diet markedly changes the gut microbial composition in mammals, whereas little is known about the impact of fat type on gut microbiome. This study was to evaluate the effects of fat sources on intestinal microbiota of nursery pigs. Eighteen pigs (28 days of age, 8.13 ± 0.10 kg BW) were housed individually (*n* = 6 per treatment) and allotted to three treatments based on a randomized complete block design. Pigs were fed basal diets with three different fat sources: 6.0% soybean oil (SBO), 6.0% palm oil (PO), and 7.5% encapsulated palm oil (EPO, contains 80% palm oil) respectively. Pigs were euthanized after 28 days of *ad libitum* feeding, and the digesta in the distal duodenum, jejunum, ileum, cecum and colon of each pig were obtained for microbial composition analysis. Correlation analyses were also performed between microbial composition with nutrients digestibility or growth performance. The results showed that pigs fed PO had marked changes in the bacteria community composition with increasing the richness and diversity in duodenum and jejunum (*P* < 0.05). Increased abundances of *Proteobacteria* in duodenum, jejunum and cecum, and decreased abundance of *Firmicutes* in jejunum were observed in pigs fed PO compared to SBO and EPO. Pigs fed EPO decreased abundances of *Proteobacteria* in duodenum and jejunum, and increased abundance of *Firmicutes* compared to pigs fed PO, and was similar to pigs fed SBO. The microbial changes (genus) had significant negative correlation with the fat digestibility. These results indicate that palm oil supplementation in nursery pig diet alters the gut microbial composition, with the most significant changes observed in small intestine. Encapsulation of palm oil, which helps increase the digestibility of palm oil, have beneficial effect on the microbial disturbance caused by palm oil supplementation. Our findings provide a better understanding of how different fat types influence microbial composition in different parts of the intestinal tract and the correlation between bacteria composition and nutrients digestibility, which may provide a new perspective for the rational application of fat in diet.

## Introduction

Dietary composition not only affects the available nutrients for hosts, but also regulates gut microbial community (Ley et al., 2006). Fat is a critical dietary nutrient, which is mainly digested and absorbed in the small intestine, such as duodenum and jejunum, rather than the large intestine (Tancharoenrat et al., 2014). The digestibility of long-chain saturated fats (palm oil, lard, tallow) is lower than that of long-chain unsaturated fats (soybean oil, corn oil) and medium-chain saturated fats (coconut oil) (Cera et al., 1988, 1989, 1990). Those indigestible fats might act as a nutritional source for bacteria and therefore have the potential to regulate gut bacterial community.

The gut microbiota is an important part of the intestinal ecosystem, which is closely related to the intestinal epithelial cell integrity, nutrient digestion and metabolism, immune response and diseases (Nicholson et al., 2005; Hooper et al., 2012). High-fat diet interferes with the balance of energy intake and expenditure by changing the gut microbial composition (KałuŻna-Czaplińska et al., 2017), and has a long-term impact on the structure of gut microbiota (Liu et al., 2018). High-fat diet increases *Firmicutes* and decreases *Bacteroidetes* in gut (De Wit et al., 2012). In addition, dietary fat composition may also have the potential to reshape the microbiota in the gut (Huang et al., 2013). For instance, variance of Bacteroides spp. is associated with the ratio of dietary saturated and unsaturated fatty acids (Lappi et al., 2013; Simoes et al., 2013). Different type and quantity of hydrolyzate of fat may have varying impacts on the microbial composition in the intestine (Fava et al., 2013). Some hydrolyzates (medium-chain fatty acids) have antibacterial activity, which could insert into the bacteria cell membrane, change the permeability of sensitive strain, and subsequently lead to leakage of intracellular protein and ion (Altieri et al., 2009). However, some hydrolyzates (glycerol, fatty acids or triglycerides) are utilized by lipolytic bacteria to produce short-chain fatty acids, which provide energy for bacteria and intestinal epithelial cells (De et al., 2018). The genus *Phascolarctobacterium* and *Anaerovibrio* are typical lipolytic bacteria that secreted extracellular esterase to break down triglycerides and its hydrolyzates (Yohei et al., 2012; De et al., 2018). Traditionally, the conventional or cultural standard methods are widely used to study the intestinal bacterial composition. However, this is a less efficient way and has potential method bias. Many of the phylotypes found in the intestine tract are detected only through the use of molecularly based methods (Rajilć Stojanović et al., 2007). Recently, the methods of 16S rRNA gene amplification and high-throughput pyrosequencing were widely used in the microbial composition analysis (Liu et al., 2018). More phylotypes of *Phascolarctobacterium* and *Anaerovibrio* were deciphered with the advance of 16S rRNA gene sequence similarities (Watanabe et al., 2012; Florence et al., 2013).

Different segments of the intestine have different physicochemical and nutritional conditions for different microbial communities (Pereira and Berry, 2017). However, it is unclear about the effect of fat types and the digestibility of different fat on microbial community in the foregut and hindgut. Previously, we reported the digestibility of fat is largely dependent on the types and sources (Yang et al., 2018). This might change existing bacterial community and introduce new bacteria. Therefore, this study was conducted to investigate the effects of different fat types on gut microbiota in the duodenum, jejunum, ileum, cecum and colon of nursery pigs using 16S rRNA gene amplification and high-throughput pyrosequencing methods, and to analyze the correlations between gut microbial composition and nutrient digestibility or growth performance.

## Materials and Methods

### Ethics Statement

All animal care and handling procedures were approved by the Institutional Animal Care and Use Committee of South China Agricultural University (SCAU-AEC-2010-0416), and consistent with the Guide for the Care and Use of Agricultural Animals in Research and Teaching (Federation of Animal Science Societies [FASS], 2010).

### Animal, Diet and Experiment Design

This study was a part of series studies designed to evaluate the effects of fat type and encapsulation on nutrient utilization and gut microbes of pigs. The detailed description of the experimental setup and encapsulation process was reported in our previous study (Yang et al., 2018). Eighteen pigs [Duroc × (Landrace × Yorkshire)] weaned at 21 days of age were allotted to three treatments based on BW and sex at 28 days of age (8.13 ± 0.10 kg BW) in a randomized complete block design (six pigs per treatment: three males and three females). Treatments were: (1) basal diet with 6.0% soybean oil (SBO, Cargill Grain & Oilseeds Ltd., Dongguan, China), (2) basal diet with 6.0% palm oil (PO, Yihai Kerry Grain & Oil Ltd., Guangzhou, China), (3) basal diet with 6.0% palm oil from encapsulated palm oil (EPO, contains 80% palm oil, 8.5% dried casein, and 11.5% whey powder). All diets were formulated to meet the nutrient requirements of 7–11 kg pigs (Supplementary Table S1; NRC, 2012). Pigs were housed individually for 4 weeks in stainless steel cages in a temperature-controlled room and allowed *ad libitum* access to feed and water throughout the experiment. Three female pigs, one from SBO, PO and EPO respectively, were removed from our experiment because of diarrhea at the end of the experiment. Pigs were euthanized by intramuscular injection of sodium pentobarbital (50 mg/kg BW, Sigma, United States) after 28 days of feeding. The digesta in the distal duodenum, jejunum, ileum, cecum and colon of each pig were collected, and then stored at −80°C until further DNA extraction.

### DNA Extraction, 16S rRNA Gene Amplification and High-Throughput Pyrosequencing

Total genomic DNA in digesta (Duodenum, Jejunum, Ileum, Cecum and Colon) was extracted from 0.25 g of sample using a PowerFecal^®^ DNA Isolation kit (MO BIO Laboratories Inc., Carlsbad, CA, United States) according to the manufacturer’s instructions. The concentration of DNA was quantified using NanoDrop spectrophotometer (Thermo Fisher Scientific Inc., Wilmington, DE, United States). The V4 region of 16S rRNA gene (from 515 to 806) was amplified with universal bacterial primers (forward 5′-GTG CCA GCM GCC GCG G-3′ and reverse 5′-GGA CTA CHV GGG TWT CTA AT-3′). PCR was performed in triplicate using a 20-μL reaction system containing 0.8 μL of each primer, 10 ng template DNA, 4 μL 5 × FastPfu buffer (TransGen Biotech, China), 2 μL 2.5 mM dNTPs, and 0.4 μL FastPfu polymerase (TransGen Biotech, China). PCR was performed on ABI GeneAmp^®^ PCR system 9700 (Applied Biosystems, Foster City, CA, United States) set as follows: 95°C for 5 min, followed by 27 cycles at 95°C for 30 s, 55°C for 30 s, and 72°C for 45 s, and a final extension at 72°C for 10 min. The PCR products from the three replicate amplifications were mixed, and electrophoresed on 2% agarose gels. Amplicons were purified using the AxyPrep DNA Gel Extraction Kit (Axygen Biosciences, Union City, CA, United States) according to the manufacturer’s instructions, and were quantified with a QuantiFluor TM-ST fluorometer (Promega, Madison, WI, United States). Equal molar ratios of purified amplicons were pooled from each sample and paired-end sequenced (2 × 250) on an Illumina Hiseq PE250 platform according to standard protocols (Caporaso et al., 2012).

### Bioinformatics Analysis

Raw sequence data were trimmed, filtered, aligned, and classified using the QIIME (Ver. 1.7.0) software package (Campbell et al., 2010). The base pair reads were truncated at any site receiving an average quality score <20 over a 10 bp sliding window, discarding the truncated reads that were shorter than 50 bp. The reads with primer mismatch numbers larger than 2 and with the barcode mismatches larger than 0 were removed, the mismatch ratio in the overlapping regions was not more than 0.2. The tags sequences obtained after the above processing were compared with the Gold database using UCHIME^1^, and the chimeras were removed to get the effective tags (Edgar et al., 2011). Operational taxonomic units (OTUs) were clustered using the average neighbor algorithm with a cutoff of 97% similarity using UPARSE (Ver. 7.0.1001)^2^. Representative sequences from each OTU were then assigned to taxa using QIIME^3^ and the Ribosomal Database Project (RDA) classifier algorithm (Cole et al., 2009) based on Silva^4^ database at an 80% confidence level. Alpha diversity such as species richness estimator (Chao1 and ACE) and diversity indices (Shannon and Simpson) were calculated using MOTHUR (Schloss et al., 2009). Unweighted Unifrac distance-based principal coordinate analysis (PCoA) based on OTUs, together with a molecular variance analysis (AMOVA), were carried out to provide an overview of the microbial response to treatments.

The 16S sequence information in this paper has been deposited in the GenBank Sequence Read Archive database under accession number SRP226668.

### Correlation Analysis

In our previous study, fat types significantly affected the growth performance and nutrient utilization (Yang et al., 2018). Pigs fed EPO had increased (*P* < 0.05) average daily gain (ADG) compared to pigs fed PO, whereas no difference was observed between pigs fed PO and SBO (268, 241 vs. 300 g/d; SBO, PO vs. EPO). The average daily feed intake (ADFI) was similar among treatments (427, 417 vs. 437 g/d; SBO, PO vs. EPO). Pigs fed PO had increased (*P* < 0.05) feed conversion ratio (ADFI: ADG, F/G) compared to pigs fed EPO, and pigs fed SBO was similar to PO and EPO (1.60, 1.74 vs. 1.50; SBO, PO vs. EPO). Pigs fed PO had increased (*P* < 0.05) diarrhea incidence (DI) compared to pigs fed SBO and EPO (10.68, 14.44 vs. 7.46; SBO, PO vs. EPO). Pigs fed EPO had increased (*P* < 0.05) apparent total tract digestibility (ATTD) of crude protein (CP) compared to pigs fed SBO, and pigs fed PO was similar to SBO and EPO (77.0%, 80.8% vs. 83.3%; SBO, PO vs. EPO). Pigs fed PO had decreased (*P* < 0.05) ATTD of crude fat (ether extract, EE) compared to pigs fed SBO and EPO (87.5%, 41.7% vs. 86.4%; SBO, PO vs. EPO).

To understand the effect of fat type on the relationship between microbial composition with growth performance or nutrient utilization, a Pearson’s correlation analysis was performed.

### Statistical Analysis

Graphing and statistical analysis were performed using GraphPad Prism (Ver. 8.0.1, La Jolla, CA, United States) and IBM SPSS Statistics software (Ver. 22.0, IBM, Armonk, NY, United States). The microbial diversity data and data of taxa richness, which had a normal distribution were analyzed by the one-way ANOVA procedures. The relative abundance of bacterial communities, which had a non-normal distribution was analyzed by the Kruskal–Wallis sum-rank test. Data were expressed as the means ± SEM. Significant differences were considered at *P* < 0.05. Correlations between bacterial abundance (genus proportion from pyrosequencing analysis) with growth performance or digestibility related indicators were analyzed by Pearson’s correlation test using SPSS. Correlation was considered significant when the absolute value of Pearson correlation coefficient was >0.5 and *P* < 0.05.

## Results

### Effects of Fat Types on Bacterial Community in Digesta Revealed by Pyrosequencing

A total of 2,819,119 V4 16S rRNA sequences were obtained from the 75 samples from 15 pigs, with an average of 37,588 sequence reads (average length of 255 bp) for each sample, which were used for subsequent analysis. The rarefaction curves tended to approach a plateau, indicating that sequencing depth was sufficient to detect the vast majority of bacteria present (Supplementary Figure S1).

**FIGURE 1:**
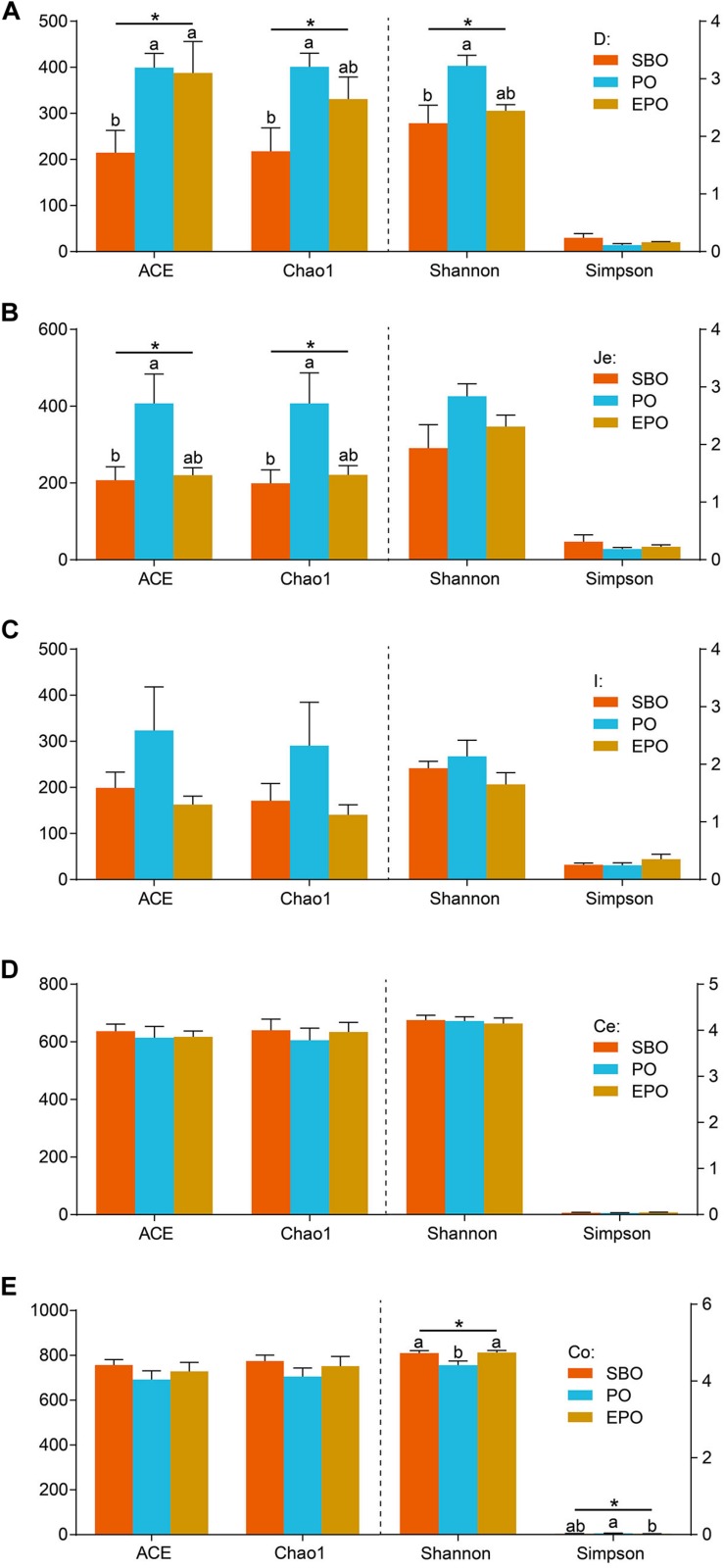
Alpha diversity including ACE, Chao1, Shannon, and Simpson of duodenum **(A)**, jejunum **(B)**, ileum **(C)**, cecum **(D),** and colon **(E)** bacterial community at the 97% similarity level in the SBO, PO, and EPO groups. The values are expressed as the means ± SEM., bars with different letters are different from each other, *P* < 0.05.

The bacterial richness and diversity at the 97% similarity level in each sample are presented in Figure 1. In the duodenum, pigs fed PO increased (*P* < 0.05) species richness and diversity indices compared with pigs fed SBO, as reflected by the ACE, Chao1 and Shannon indices. Pigs fed SBO and PO performed significant different species richness and diversity, but EPO was similar to SBO and PO (Figure 1A). In the jejunum, pigs fed PO increased (*P* < 0.05) species richness indices (ACE and Chao 1) compared with pigs fed SBO, whereas pigs fed EPO had similar ACE and Chao 1 indices compared with pigs fed SBO and PO. Species diversity indices (Shannon and Simpson) in the jejunum were not affected by different fat supplementation (Figure 1B). In the ileum and cecum, species richness and diversity indices were not affected by the dietary treatments (Figures 1C,D). In the colon, species diversity indices (Shannon and Simpson) of pigs fed PO were different from pigs fed SBO and EPO, whereas species richness indices were not affected by the dietary treatments (Figure 1E).

**FIGURE 2:**
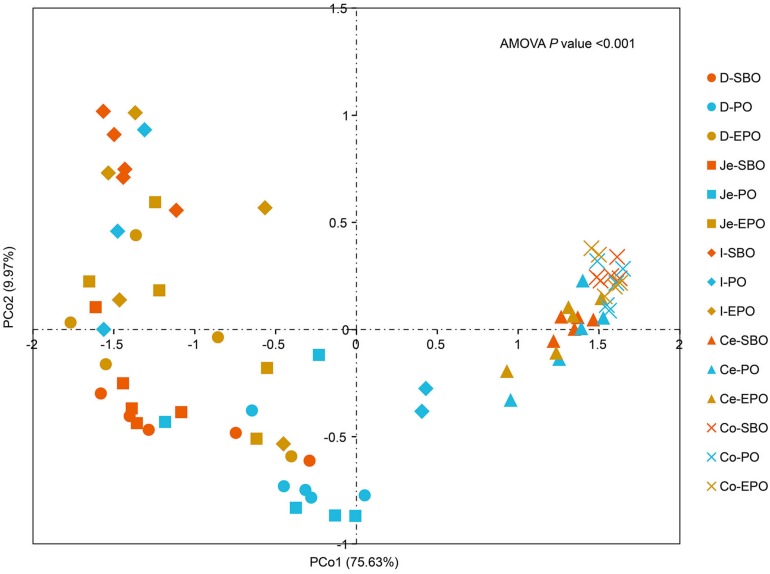
Principle coordinate analysis (PCoA) of gut microbiota in the SBO, PO, and EPO groups by unweighted UniFrac distance (*n* = 5 per group). The percentage of variation explained by PCo1 and PCo2 are indicated in the axis. Abbreviation: D-, duodenum-; Je-, jejunum-; I-, ileum; Ce-, cecum-; Co-, colon; SBO, soybean oil; PO, palm oil; EPO, encapsulated palm oil.

Principal coordinate analysis was performed based on unweighted Unifrac distance to reveal the impact of different fat supplementation on the microbiota composition (Figure 2), which indicated a distinct separation in microbiota composition in the small intestine (AMOVA, *P* < 0.001). Samples were clustered primarily by the gut segment (small intestine/large intestine). AMOVA analysis was carried out to further evaluate the statistical significance of the spatial separation among different groups in PCoA plots (Supplementary Table S2). There was a significant difference in the microbiota composition of pigs fed SBO, PO, and EPO in duodenum (*P* = 0.003, between SBO and PO; *P* = 0.038, between SBO and EPO; *P* = 0.013, between PO and EPO; Supplementary Table S2). Pigs fed SBO had significant dissimilarities compared to pigs fed PO in jejunum (*P* = 0.013) and ileum (*P* = 0.034).

**FIGURE 3:**
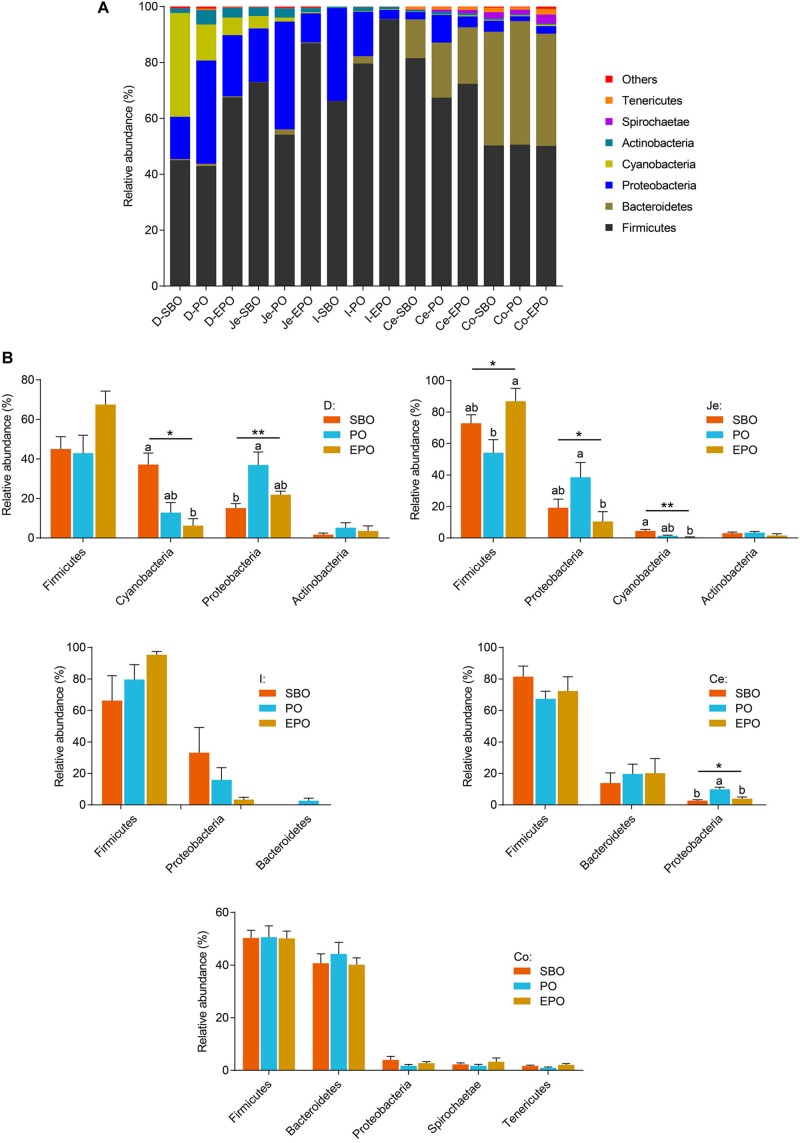
Phylum-level composition. **(A)** The phylum -level taxonomic composition of average relative abundance of bacterial communities in the SBO, PO, and EPO groups. **(B)** The changed bacterial phyla found in each segment of the intestine of piglets (the mean abundance more than 2% at least one group, analyzed using Kruskal–Wallis sum-rank test). *P < 0.05; **P < 0.01. D-, duodenum-; Je-, jejunum-; I-, ileum; Ce-, cecum-; Co-, colon; SBO, soybean oil; PO, palm oil; EPO, encapsulated palm oil.

At phylum level, *Firmicutes*, *Proteobacteria*, and *Cyanobacteria* were the dominant phyla in duodenum, *Firmicutes* and *Proteobacteria* were the dominant phyla in ileum and jejunum, *Firmicutes* and *Bacteroidetes* were the most detected phyla in cecum and colon (Figure 3A). In duodenum and jejunum (Figure 3B), pigs fed PO had the greatest relative abundance of bacteria belonging to the phyla *Proteobacteria*, and pigs fed SBO had increased (*P* < 0.05) abundance of bacteria from the phyla *Cyanobacteria* compared with pigs fed EPO. Pigs fed EPO increased the abundance of bacteria from the phyla *Firmicutes* compared with pigs fed PO (*P* < 0.05). However, no significant changes in the abundance of bacteria in phylum were observed in ileum, cecum and colon, except that pigs fed PO had increased (*P* < 0.05) abundance of the bacteria belonging to the phyla *Proteobacteria* compared with pigs fed SBO and EPO in cecum.

**FIGURE 4:**
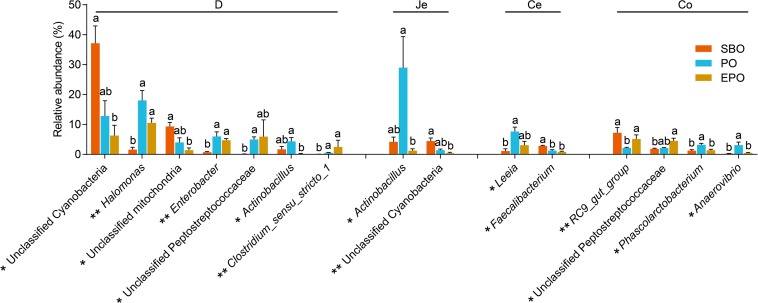
The significantly changed genera found in each segment of intestine of piglets (the mean abundance more than 2% at least one group, analyzed using Kruskal Wallis sum-rank test). *P < 0.05; **P < 0.01. D-, duodenum-; Je-, jejunum-; Ce-, cecum-; Co-, colon; SBO, soybean oil; PO, palm oil; EPO, encapsulated palm oil.

The 44 most abundant genera (the mean abundance more than 2% at least one group) are listed in Supplementary Figure S2. In duodenum, pigs fed PO and EPO increased (*P* < 0.05) the abundance of *Halomonas*, *Enterobacter*, unclassified peptostreptococcaceae and *Clostridium sensu stricto 1* compared with pigs fed SBO (Figure 4). In duodenum and jejunum, pigs fed SBO had increased (*P* < 0.05) abundance of unclassified cyanobacteria compared to pigs fed EPO, and pigs fed EPO had decreased abundance (*P* < 0.05) of *Actinobacillus* compared to pigs fed PO. In cecum, pigs fed PO had increased (*P* < 0.05) abundance of *Leeia* compared to pigs fed SBO, and pigs fed SBO had increased (*P* < 0.05) abundance of *Faecalibacterium* compared to PO and EPO. In colon, pigs fed PO had decreased (*P* < 0.05) abundance of *RC9 gut group*, and increased (*P* < 0.05) abundance of *Phascolarctobacterium* and *Anaerovibrio* compared to pigs fed SBO and EPO (Figure 4). However, no significant changes in the abundance of genera were observed among treatment groups in ileum.

### Correlations Between Microbial Composition With Growth Performance or Nutrient Digestibility

**FIGURE 5:**
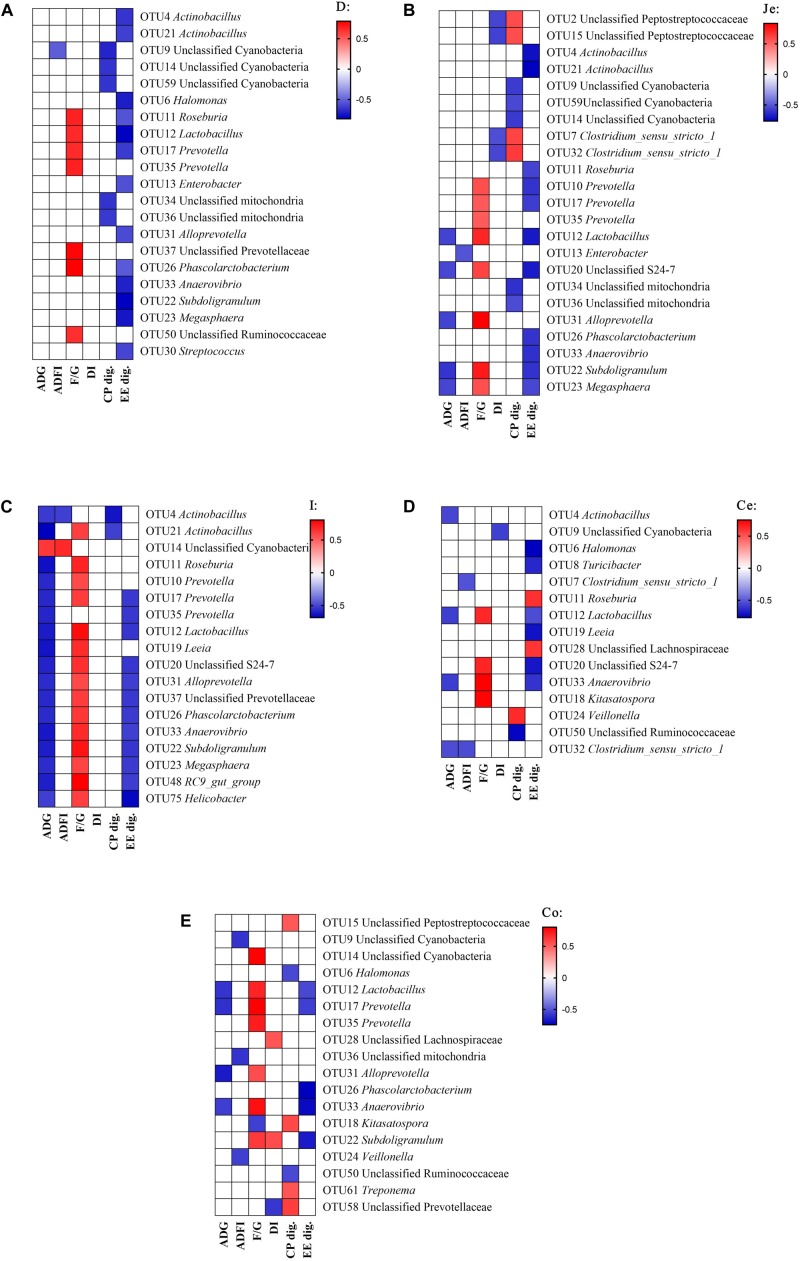
Correlation analysis between the abundance of microbiota (genus) and phenotypic data in the gut. Correlation was considered significant when the absolute value of Pearson correlation coefficient was > 0.5 and *P* < 0.05. **(A)** duodenum, **(B)** jejunum, **(C)** ileum, **(D)** cecum, **(E)** colon; ADG, average daily gain; ADFI, average daily feed intake; F/G, Feed conversion ratio; DI, diarrhea incidence; CP dig., crude protein digestibility; EE dig., crude fat digestibility.

Pearson’s correlation analyses were performed between microbial composition (genus) with growth performance indicators or digestibility of CP and EE, in order to understand the relationships between the microbial composition changes with the change in growth performance or nutrient utilization among different fat sources (Figure 5). In the duodenum (Figure 5A), there were significantly correlations between the digestibility and bacteria, the EE digestibility was negatively correlated with the abundance of *Actinobacillus*, *Halomonas*, *Roseburia*, *Lactobacillus*, *Prevotella, Enterobacter*, *Alloprevotella*, *Phascolarctobacterium*, *Anaerovibrio*, *Subdoligranulum*, *Megasphaera* and *Streptococcus*, and CP digestibility was negatively correlated with the abundance of unclassified Cyanobacteria and unclassified mitochondria. For growth performance, the feed conversion ratio (F/G) was positively correlated with the abundance of *Roseburia*, *Lactobacillus*, *Prevotella*, unclassified Prevotellaceae, *Phascolarctobacterium* and unclassified Ruminococcaceae.

In the jejunum (Figure 5B), the EE digestibility was negatively correlated with the abundance of *Actinobacillus*, *Roseburia*, *Lactobacillus*, *Prevotella*, Unclassified S24-7, *Phascolarctobacterium*, *Anaerovibrio*, *Subdoligranulum*, and *Megasphaera*. The CP digestibility was negatively correlated with the abundance of unclassified Cyanobacteria, and positively correlated with the abundance of unclassified Peptostreptococcaceae and *Clostridium_sensu_stricto_1*. For growth performance, the diarrhea incidence (DI) was negatively correlated with the abundance of unclassified Peptostreptococcaceae and *Clostridium_sensu_stricto_1*, the F/G was positively correlated with the abundance of *Lactobacillus*, *Prevotella*, Unclassified S24-7, *Alloprevotella*, *Subdoligranulum*, and *Megasphaera*, and the ADG was negatively correlated with the abundance of *Lactobacillus*, Unclassified S24-7, *Alloprevotella*, *Subdoligranulum* and *Megasphaera*.

In the ileum (Figure 5C), the EE digestibility was negatively correlated with the abundance of *Prevotella, Lactobacillus*, Unclassified S24-7, *Alloprevotella*, unclassified Prevotellaceae, *Phascolarctobacterium*, *Anaerovibrio*, *Subdoligranulum*, *Megasphaera*, *RC9 gut group*, and *Helicobacter*. The CP digestibility was negatively correlated with the abundance of *Actinobacillus*. For growth performance, the F/G was positively correlated with the abundance of *Actinobacillus*, *Roseburia*, *Lactobacillus*, *Prevotella, Leeia*, Unclassified S24-7, *Alloprevotella*, unclassified Prevotellaceae, *Phascolarctobacterium*, *Anaerovibrio*, *Subdoligranulum*, *Megasphaera*, *RC9 gut group*, and *Helicobacter*. The ADG was negatively correlated with the abundance of *Actinobacillus*, *Roseburia*, *Lactobacillus*, *Prevotella, Leeia*, Unclassified S24-7, *Alloprevotella*, unclassified Prevotellaceae, *Phascolarctobacterium*, *Anaerovibrio*, *Subdoligranulum*, *Megasphaera*, *RC9 gut group*, and *Helicobacter*, and was positively correlated with the abundance of unclassified Cyanobacteria.

In the cecum (Figure 5D), the EE digestibility was negatively correlated with the abundance of *Halomonas*, *Turicibacter*, *Lactobacillus*, *Leeia*, Unclassified S24-7, and *Anaerovibrio*, and was positively correlated with the abundance of *Roseburia* and unclassified Lachnospiraceae. The CP digestibility was negatively correlated with the abundance of unclassified Ruminococcaccae, and was positively correlated with the abundance of *Veillonella*. For growth performance, the DI was negatively correlated with the abundance of unclassified Cynobacteria. The F/G positively correlated with the abundance of *Lactobacillus*, Unclassified S24-7, *Anaerovibrio*, and *Kitasatospora*. The ADG was negatively correlated with the abundance of *Actinobacillus*, *Lactobacillus*, *Anaerovibrio* and *Clostridium_sensu_stricto_1.*

In the colon (Figure 5E), the EE digestibility was negatively correlated with the abundance of *Lactobacillus*, *Prevotella, Phascolarctobacterium*, *Anaerovibrio* and *Subdoligranulum*. The CP digestibility was negatively correlated with the abundance of *Halomonas* and unclassified Ruminococcaceae, and was positively correlated with the abundance of unclassified Peptostreptococcaceae, *Kitasatospora*, *Treponema* and unclassified Prevotellaceae. For growth performance, the DI was negatively correlated with the abundance of unclassified Prevotellaceae, and was positively correlated with the abundance of unclassified Lachnospiraceae and *Subdoligranulum*. The F/G was positively correlated with the abundance of unclassified Cyanobacteria, *Lactobacillus*, *Prevotella, Alloprevotella*, *Anaerovibrio*, and *Subdoligranulum*, and was negatively correlated with the abundance of *Kitasatospora*. The ADG was negatively correlated with the abundance of *Lactobacillus*, *Prevotella, Alloprevotella* and *Anaerovibrio*.

## Discussion

Fat is one of the three most important nutrients in human and animal foods. Different types of dietary fat have great impact on gut microbial composition in mammals (Fava et al., 2013). In this study, we investigated the changes of microbial community in the duodenum, jejunum, ileum, cecum and colon digesta of nursery pigs in response to different fat sources using high-throughput sequencing. Fat types markedly affected microbial composition in duodenum and jejunum, with less effect in ileum, cecum, and colon. The microbial changes were significantly correlated with the fat digestibility. These findings indicate that the differences in fat digestibility caused by fat sources significant influence intestinal microbial community.

### Fat Types Induced the Microbial Changes in Nursery Pigs, With More Evident Effect Observed in the Small Intestine

Different segments of the intestine have different physicochemical and nutrient conditions for different microbial communities (Pereira and Berry, 2017). In this study, the impacts of fat types on the microbial composition in the small intestine and large intestine were varying, with marked changes in duodenum and jejunum, and less effect in cecum and colon, which was confirmed by the Alpha diversity, PCoA and AMOVA analyses of microbiota (Figures 1, 2 and Supplementary Table S2). The digestion and absorption of fat occurs in the small intestine with the majority of fat and fatty acids digested and absorbed in duodenum and jejunum, the large intestine does not participate in the digestion of fat (Tancharoenrat et al., 2014). Therefore, the different fat digestibility along the gut may account for the difference of the microbiota in the small and large intestine. In our previous study, different fat sources had varying digestibility, in which pigs fed PO decreased digestibility of crude fat (ether extract, EE) compared with pigs fed SBO and EPO (87.5%, 41.7% vs. 86.4%; SBO, PO vs. EPO). The type and quantity of hydrolyzate of fat may have varying impacts on the microbial composition in the same segment of intestine (Fava et al., 2013), and parts of the fatty acids and monoglycerides hydrolyzed from fat (triglyceride) have antibacterial activity (Altieri et al., 2009). The different hydrolyzates of soybean oil and palm oil, which have different fatty acid profile, may induce varying responses of microbial composition. In our study, the different microbial composition in PO and EPO may be caused by the varying quantities of hydrolyzates.

At phylum level, pigs fed PO markedly increased the phyla *Proteobacteria* in duodenum, jejunum and cecum, and reduced the phyla *Firmicutes* in jejunum in the present study. The phyla *Proteobacteria* is the dominant phyla in small intestine, including many kinds of pathogenic bacteria, which are intricately involved in the luminal dysbiosis caused by inflammatory bowel disease (IBD) (Mukhopadhya et al., 2012). On the contrary, *Firmicutes*, which is the most predominant phyla in the intestine, contains the vast majority of potentially beneficial species. It is reported that the depletion of *Faecalibacterium*, belonging to the *Firmicutes* phylum, has been associated with IBD (Marchesi et al., 2016). Therefore, supplementation of PO in nursery pig diet may induce a breakdown in the balance of putative pathogenic bacteria and protective commensal bacteria, and EPO supplementation increased the digestibility of palm oil, which may have beneficial effect on the balance of those intestinal microbes (Figure 3B).

### Fat Types Induced Specific Changes in Microbial Genera and Its Correlations With Nutrients Digestibility or Growth Performance

With specific genera, pigs fed PO increased the genus *Actinobacillus* in duodenum and jejunum, and the abundance of *Actinobacillus* in duodenum and jejunum was negatively correlated with the EE digestibility. Some species within the genera *Actinobacillus* are putative pathogenic bacteria in association with clinical condition of the respiratory system (Teshima et al., 2017; To et al., 2017). In the present study, the low digestibility of palm oil significantly increased the abundance of *Actinobacillus*, whereas increased digestibility of palm oil by encapsulation reduced the abundance of *Actinobacillus* in the small intestine. The phyla *Cyanobacteria* is well known as oxygenic photosynthetic bacteria in a wide variety of ecological environments that enhances nitrogen fixation (Liberton et al., 2019). The survival of *Cyanobacteria* during gut passage may enhance nutrient availability (Friedland et al., 2005). In our study, pigs fed palm oil reduced the abundance of unclassified cyanobacteria in duodenum and jejunum, which was negatively correlated with the CP digestibility. Therefore, the protein in digesta may promote the growth of *Cyanobacteria*. The abundance of *Clostridium sensu stricto 1* and unclassified peptostreptococcaceae (belongs to *Firmicutes*) were increased in duodenum of pigs fed PO and EPO, with the same tendency in jejunum. It has been reported that the proportion of *Clostridium sensu stricto 1* was significantly decreased when pigs fed low protein diet (Fan et al., 2017), and similar results was also observed in rats (Zhu et al., 2017). In the present study, the abundance of *Clostridium sensu stricto 1* and unclassified peptostreptococcaceae were positively correlated with the CP digestibility. The abundance of bacteria may be affected by the amount of protein hydrolyzate.

In addition, the abundance of *Phascolarctobacterium* and *Anaerovibrio* were negatively correlated with the EE digestibility in duodenum, jejunum, ileum and colon (Figure 5). The genus *Anaerovibrio* is a typical lipolytic bacteria that hydrolyzes triglycerides to produce glycerol and fatty acids, and ferments glycerol to produce propionic and succinic acids (Schauder and Schink, 1989; De et al., 2018). The genus *Phascolarctobacterium* has the ability to ferment glycerol into acetate and propionate in the human gut (Yohei et al., 2012; Wu et al., 2017). The increased residual fat in digesta of nursery pigs may promote the proliferation of *Phascolarctobacterium* and *Anaerovibrio*. Likewise, the abundance of some genera had negative correlation with the digestibility of crude fat, such as *Halomonas*, *Enterobacter, Prevotella, Alloprevotella, Subdoligranulum, Leeia*, which all belong to putative pathogenic bacteria that induce mortality and intestinal microbiota disturbance.

The correlation between the abundance of bacteria and growth performance mainly reflected in the influence of feed conversion ratio (F/G) in this study. The proliferation of potentially pathogenic bacteria in the gut may lead to increased levels of endotoxin secreted by the bacteria (Marchesi et al., 2016), and then affect the health and function of intestinal epithelial cells (Mani et al., 2013), consequently, result in disrupted digestion and absorption of nutrients and poor feed conversion efficiency.

## Conclusion

The present study demonstrated that palm oil supplementation in nursery pig diet altered the gut microbial composition by increasing the abundance of *Proteobacteria* while reducing the abundance of *Firmicutes*, with more evident effect observed in the small intestine. The microbial changes were negatively correlated with fat digestibility, whereas encapsulation of palm oil (EPO) increased the digestibility of palm oil, which may have beneficial effect on the intestinal microbes. The findings provide evidence that different fat types may affect fat digestibility and further influence composition of gut microbiome.

## Data Availability Statement

The datasets generated for this study can be found in the GenBank Sequence Read Archive database, accession number SRP226668.

## Ethics Statement

The animal study was reviewed and approved by Institutional Animal Care and Use Committee of South China Agricultural University.

## Author Contributions

WG, SZ, and FC conceived and designed the whole trial. FY and MT conducted the pig trial. FY and JC conducted the laboratory analyses. FY, SZ, and WG wrote the manuscript. All authors agreed to be accountable for all aspects of the work.

## Conflict of Interest

The authors declare that the research was conducted in the absence of any commercial or financial relationships that could be construed as a potential conflict of interest.

## Footnotes

1 http://www.drive5.com/usearch/manual/uchime_algo.html

2 http://drive5.com/uparse/

3 http://qiime.org/scripts/assign_taxonomy.html

4 http://www.arb-silva.de

## Supplementary Material

FIGURE S1 Rarefaction curves of duodenum **(A)**, jejunum **(B)**, ileum **(C)**, cecum **(D)** and colon **(E)** samples comparing the number of sequences with the number of OTUs from the microbiota in the digesta of piglets in each treatment group.

FIGURE S2 The relative abundance of the most abundant genera 44 OTUs with a significant difference (the mean abundance more than 2% at least one group) in the SBO, PO, and EPO groups. (analyzed using Kruskal–Wallis sum-rank test) ^∗^*P* < 0.05; ^∗∗^*P* < 0.01. D-, duodenum-; Je-, jejunum-; I-, ileum; Ce-, cecum-; Co-, colon; SBO, soybean oil; PO, palm oil; EPO, encapsulated palm oil.

TABLE S1 Composition and nutrient content of experimental diets (as-fed basis).

TABLE S2 Further evaluation of the statistical significance of the spatial separation among the different groups in PCoA plots using AMOVA analysis (*P*-value).

## Abbreviations

ADFI: average daily feed intake
ADG: average daily gain
AMOVA: a molecular variance analysis
ATTD: apparent total tract digestibility
BW: body weight
CP: crude protein
DI: diarrhea incidence
DM: dry matter
EE: ether extract
EPO: encapsulated palm oil
F/G, feed conversion ratio: ADFI/ADG
OTUs: operational taxonomic units
PCoA: principal coordinates analysis
PO: palm oil
SBO: soybean oil
SEM: standard error mean.
