# Supplementary figures and images for: Different Sources of High Fat Diet Induces Marked Changes in Gut Microbiota of Nursery Pigs
Source: Front Microbiol. 2020 May 7;11:859. doi: 10.3389/fmicb.2020.00859 (PMC7221029; doi:10.3389/fmicb.2020.00859)

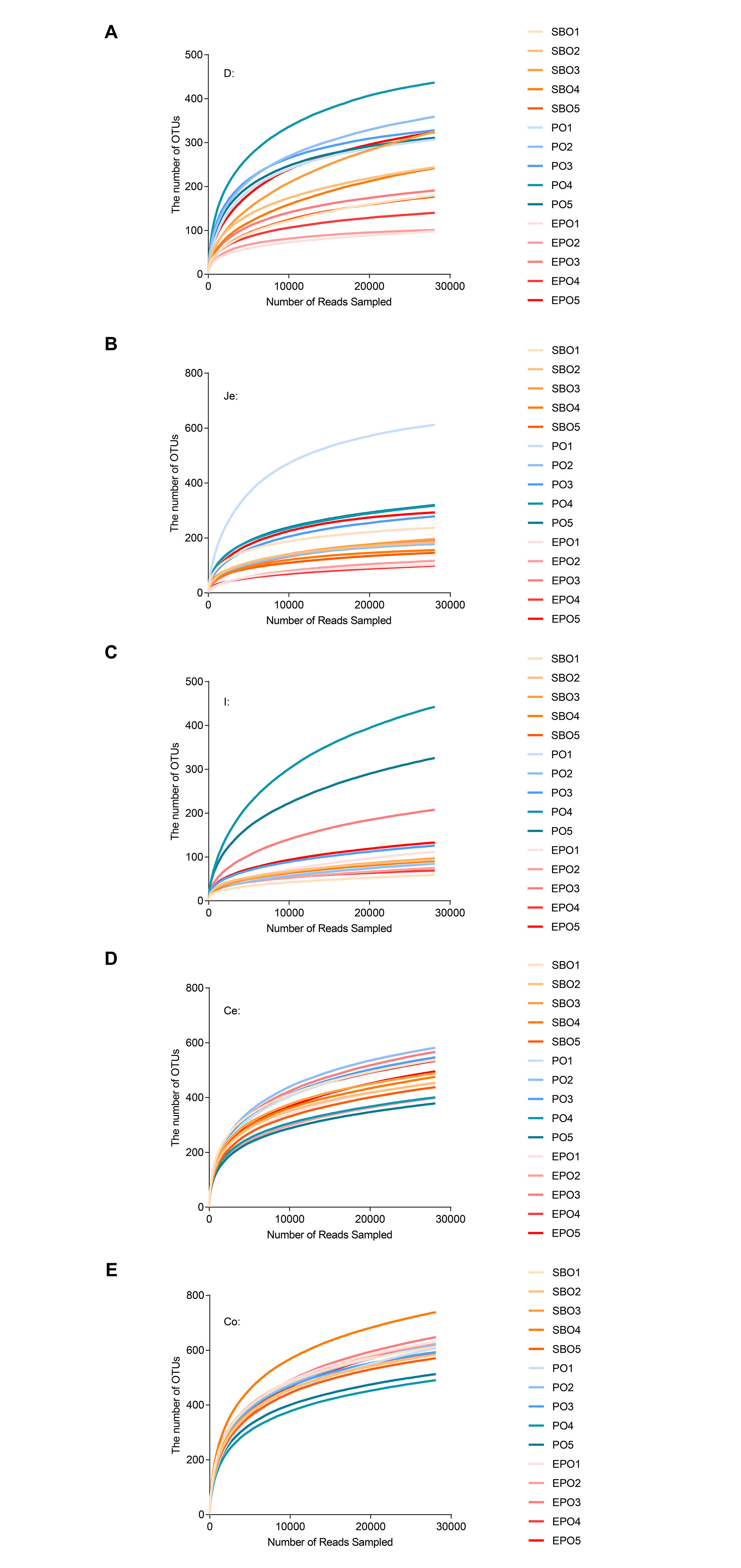

Supplement: FIGURE S1 — Rarefaction curves of duodenum (A), jejunum (B), ileum (C), cecum (D) and colon (E) samples comparing the number of sequences with the number of OTUs from the microbiota in the digesta of piglets in each treatment group. [file Image_1.jpg]

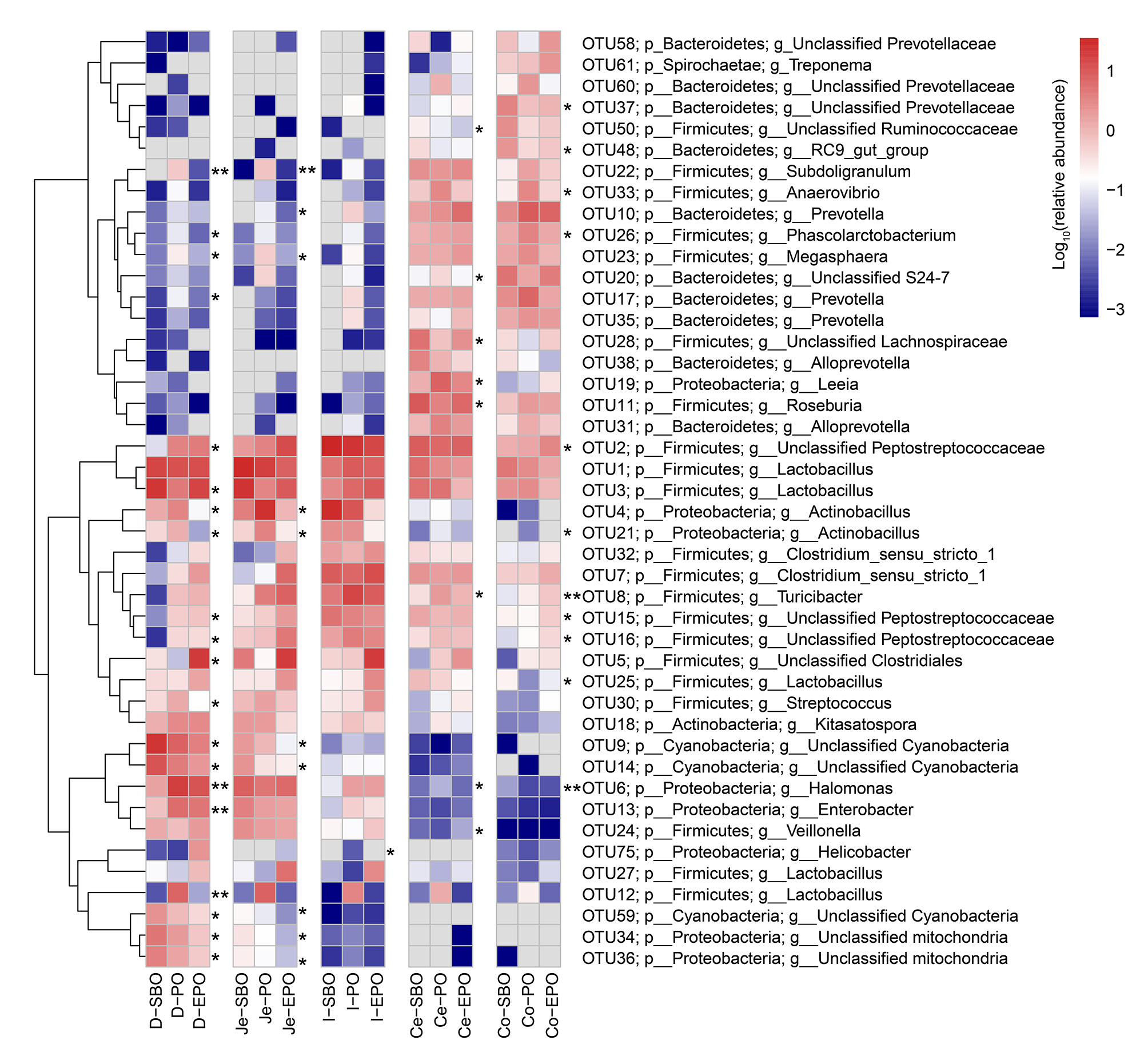

Supplement: FIGURE S2 — The relative abundance of the most abundant genera 44 OTUs with a significant difference (the mean abundance more than 2% at least one group) in the SBO, PO, and EPO groups. (analyzed using Kruskal–Wallis sum-rank test) ∗P < 0.05; ∗∗P < 0.01. D-, duodenum-; Je-, jejunum-; I-, ileum; Ce-, cecum-; Co-, colon; SBO, soybean oil; PO, palm oil; EPO, encapsulated palm oil. [file Image_2.JPEG]
