# Supplementary material for: Different Sources of High Fat Diet Induces Marked Changes in Gut Microbiota of Nursery Pigs
Source: Front Microbiol. 2020 May 7;11:859. doi: 10.3389/fmicb.2020.00859 (PMC7221029; doi:10.3389/fmicb.2020.00859)
Supplement: TABLE S1 — Composition and nutrient content of experimental diets (as-fed basis). [file Table_1.DOCX]

**Table S1** Composition and nutrient content of experimental diets (as-fed basis)

|  | Treatments | | | |
| --- | --- | --- | --- | --- |
| Item | Soybean oil | Palm oil | Encapsulated palm oil^1^ |  |
| Ingredient, % |  |  |  |  |
| Corn | 62.30 | 62.30 | 62.30 |  |
| Soybean meal, 43%CP | 4.20 | 4.20 | 4.20 |  |
| Whey powder, 3.8%CP | 5.86 | 5.86 | 5.00 |  |
| Fish meal, 62%CP | 3.50 | 3.50 | 3.50 |  |
| Soybean oil | 6.00 |  |  |  |
| Palm oil |  | 6.00 |  |  |
| Encapsulated palm oil |  |  | 7.50 |  |
| Fermented soybean meal | 12.00 | 12.00 | 12.00 |  |
| Dried casein | 0.64 | 0.64 |  |  |
| Acidifier | 0.60 | 0.60 | 0.60 |  |
| Dicalcium phosphate | 0.45 | 0.45 | 0.45 |  |
| Calcium formate | 1.34 | 1.34 | 1.34 |  |
| Salt | 0.30 | 0.30 | 0.30 |  |
| L-lysine HCL（78.8%） | 0.68 | 0.68 | 0.68 |  |
| DL-methionine（99%） | 0.37 | 0.37 | 0.37 |  |
| L-threonine（99%） | 0.31 | 0.31 | 0.31 |  |
| L-tryptophan（98.5%） | 0.08 | 0.08 | 0.08 |  |
| Vitamin premix^2^ | 0.05 | 0.05 | 0.05 |  |
| Trace element premix^3^ | 0.10 | 0.10 | 0.10 |  |
| Choline chloride 50% | 0.16 | 0.16 | 0.16 |  |
| Others^4^ | 0.71 | 0.71 | 0.71 |  |
| Total | 100.00 | 100.00 | 100.00 |  |
| Calculated nutrient composition^5^ |  |  |  |  |
| DE, Mcal/kg | 3.63 | 3.59 | 3.60 |  |
| CP, % | 17.01 | 17.01 | 17.01 |  |
| Ether Extract, % | 8.43 | 8.43 | 8.43 |  |
| Ash, % | 3.52 | 3.52 | 3.52 |  |
| Ca, % | 0.70 | 0.70 | 0.70 |  |
| P, % | 0.48 | 0.48 | 0.48 |  |
| AP, % | 0.33 | 0.33 | 0.33 |  |
| Total lysine, % | 1.40 | 1.40 | 1.40 |  |
| SID Lys, % | 1.25 | 1.25 | 1.25 |  |
| SID Met+Cys, % | 0.76 | 0.76 | 0.76 |  |
| SID Thr, % | 0.81 | 0.81 | 0.81 |  |
| SID Trp, % | 0.23 | 0.23 | 0.23 |  |
| Analyzed composition |  |  |  |  |
| GE, Mcal/kg | 4.40 | 4.58 | 4.50 |  |
| CP, % | 17.05 | 17.12 | 17.20 |  |
| Ether extract, % | 8.01 | 8.68 | 8.40 |  |

^1^The diet contains 6.0% palm oil from encapsulated palm oil (containing 80% ether extract), which were produced by combing palm oil with dried casein and whey powder in a spray-drying process causing the milk proteins to encapsulate the fat particles as they dried. Dried casein and whey powder were obtained from the same source, other treatment diets without encapsulated fat contained identical amounts of casein and whey.

^2^Provided the following amounts of vitamins per kilogram of diet: vitamin A, 12,000 IU as retinyl acetate; vitamin D3, 3,600 IU as cholecalciferol; vitamin E, 150 IU as DL-α-tocopherol acetate; vitamin K3, 7.2 mg as menadione; thiamine, 3 mg; riboflavin, 10.8 mg; pyridoxine, 5.4 mg; vitamin B12, 0.06 mg; pantothenic acid, 36.0 mg; niacin, 60.0 mg; folic acid, 6 mg; biotin, 0.6 mg.

^3^Provided the following amounts of trace minerals per kilogram of diet: Cu, 10 mg as CuSO_4_.5H_2_O; Fe, 39 mg as FeSO_4_. H_2_O; Mn, 30 mg as MnSO_4_; Zn, 39 mg as ZnSO_4_; Se, 0.15 mg as Na_2_SeO_3_; I, 0.14 mg as Ca(IO_3_)_2_; Co, 0.1 mg as CoCL_2_.

^4^Others contains antibiotics, enzymes, mycotoxin removal agent, sweetening agents, zeolite (carrier).

^5^Based on nutrient composition of feed ingredients according to NRC (2012).
