# Supplementary material for: Different Sources of High Fat Diet Induces Marked Changes in Gut Microbiota of Nursery Pigs
Source: Front Microbiol. 2020 May 7;11:859. doi: 10.3389/fmicb.2020.00859 (PMC7221029; doi:10.3389/fmicb.2020.00859)
Supplement: TABLE S2 — Further evaluation of the statistical significance of the spatial separation among the different groups in PCoA plots using AMOVA analysis (P-value). [file Table_2.DOCX]

**Table S2** Further evaluation of the statistical significance of the spatial separation among the different groups in PCoA plots using AMOVA analysis (*P*-value)

| *P*-value | D-SBO | D-PO | D-EPO |
| --- | --- | --- | --- |
| D-SBO |  |  |  |
| D-PO | 0.003 |  |  |
| D-EPO | 0.038 | 0.013 |  |

| *P*-value | Je-SBO | Je-PO | Je-EPO |
| --- | --- | --- | --- |
| Je-SBO |  |  |  |
| Je-PO | 0.013 |  |  |
| Je-EPO | 0.282 | 0.053 |  |

| *P*-value | I-SBO | I-PO | I-EPO |
| --- | --- | --- | --- |
| I-SBO |  |  |  |
| I-PO | 0.034 |  |  |
| I-EPO | 0.101 | 0.449 |  |

| *P*-value | Ce-SBO | Ce-PO | Ce-EPO |
| --- | --- | --- | --- |
| Ce-SBO |  |  |  |
| Ce-PO | 0.154 |  |  |
| Ce-EPO | 0.275 | 0.514 |  |

| *P*-value | Co-SBO | Co-PO | Co-EPO |
| --- | --- | --- | --- |
| Co-SBO |  |  |  |
| Co-PO | 0.130 |  |  |
| Co-EPO | 0.444 | 0.332 |  |

Abbreviation: D-, duodenum-; Je-, jejunum-; I-, ileum; Ce-, cecum-; Co-, colon; SBO, soybean oil; PO, palm oil; EPO, encapsulated palm oil.
